# Molecular mechanism of male differentiation is conserved in the *SRY*-absent mammal, *Tokudaia osimensis*

**DOI:** 10.1038/srep32874

**Published:** 2016-09-09

**Authors:** Tomofumi Otake, Asato Kuroiwa

**Affiliations:** 1 Functional Genome Science Biosystems Science Course, Graduate School of Life Science, Hokkaido University, Kita 10 Nishi 8, Kita-ku, Sapporo, Hokkaido 060-0810, Japan; 2 Division of Reproductive and Developmental Biology, Department of Biological Sciences, Faculty of Science, Hokkaido University, Kita 10 Nishi 8, Kita-ku, Sapporo, Hokkaido 060-0810, Japan

## Abstract

The sex-determining gene *SRY* induces *SOX9* expression in the testes of eutherian mammals via two pathways. *SRY* binds to testis-specific enhancer of *Sox*9 (TESCO) with SF1 to activate *SOX9* transcription. *SRY* also up-regulates *ER71* expression, and ER71 activates *Sox9* transcription. After the initiation of testis differentiation, SOX9 enhances *Amh* expression by binding to its promoter with SF1. SOX8, SOX9 and SOX10, members of the *SOXE* gene family, also enhance the activities of the *Amh* promoter and TESCO. In this study, we investigated the regulation of these sexual differentiation genes in *Tokudaia osimensis*, which lacks a Y chromosome and the *SRY* gene. The activity of the *AMH* promoter was stimulated by *SOXE* genes and SF1. Mutant *AMH* promoters, with mutations in its SOX and SF1 binding sites, did not show significant activity by SOX9 and SF1. These results indicate that *AMH* expression was regulated by the binding of SOX9 and SF1. By contrast, *SOXE* genes could not enhance TESCO activity. These results indicate that TESCO enhancer activity was lost in this species. Furthermore, the activity of the *SOX9* promoter was enhanced by ER71, indicating that *ER71* may play an important role in the testis-specific expression of *SOX9*.

The master sex-determining gene *SRY* (sex-determining region Y) located on the Y chromosome is present in most eutherian mammals^1^,^2^. *SRY* initiates the transcription of *SOX9 (SRY*-box 9) in the genital ridge of the XY embryo, and an up-regulation of *SOX9* expression gives rise to the Sertoli cells, resulting in testis development^3^. *Sox9/SOX9* is necessary and sufficient for male sex determination in the mouse and human. In the mouse, *SRY* activates the testis-specific expression of *Sox9* via two pathways. In the first mechanism, *SRY* binds to the enhancer TESCO (TES [testis-specific enhancer of *Sox*9] COre), which is located 13 kb upstream of *Sox9* together with SF1 (also known as nuclear receptor subfamily 5, group A, member1, NR5A1), to induce *Sox9* expression^4^. The TESCO sequence contains several *SRY* binding sites (BSs) and SF1 BSs that are highly conserved between the mouse, rat, dog, and human^4^. *Sry* expression is restricted to 10.5 and 12.5 days post-coitum (dpc) in the mouse^5^,^6^,^7^. Thereafter, SOX9 binds to *SRY* BSs in TESCO for its self-regulation^4^. In the second mechanism, *SRY* regulates *Sox9* expression via *Er71* (ETS related 71; also known as *ETS* variant 2, *ETV2*)^8^. *SRY* binds to the promoter region of *Er71* with the transcriptional factor SP1 activates *Er71* expression in the testes. ER71 subsequently regulates *Sox9* expression by binding to the *Sox9* proximal promoter. After the *Sry* expression, SOX9 binds to the *Er71* promoter to control the expression of *Er71*. Thus, transcription of *Er71* and *Sox9* are co-regulated each other in the mouse^9^.

SOX9 directly regulates the expression of *AMH* (anti-Müllerian hormone; also known as Müllerian inhibitory substance, MIS). After the initiation of testes differentiation, *AMH* expression is induced in the Sertoli cells of eutherian mammals^10^,^11^,^12^. A previous study reported that approximately 370 bp of the *Amh* 5′ flanking region was essential for its expression from 12.5 dpc until an early postnatal stage in the male mouse^13^. This region, defined as the *Amh* proximal promoter, contains one SOX BS, two SF1 BSs, one GATA4 BS, and one WT1 BS^14^,^15^,^16^,^17^,^18^,^19^,^20^,^21^. The SOX BS is the most important region for *Amh* expression^22^. Furthermore, these BSs within the *AMH* promoter are conserved in several eutherian mammals and marsupials such as the wallaby, suggesting therians (eutherians and marsupials) share a common *AMH* regulatory mechanism^23^,^24^.

Other *SOX* genes might also have important functions in testicular differentiation. The *SOX* gene family consists of 20 members. They contain a HMG (high-mobility-group) domain that binds DNA^25^, and *SOX* genes are categorized into ten subgroups^26^. Among these, *SOX8 (SRY*-box 8), SOX*9,* and *SOX10 (SRY*-box 10) belong to the SOXE group. All *SOXE* genes are expressed during mammalian testis development^25^,^27^,^28^, and the structure of these proteins is highly conserved. *In vitro* studies demonstrate that SOX8 and SOX10 can stimulate the activities of the *AMH* promoter and TESCO such as SOX9^27^,^29^. In addition, SOX8 regulates Sertoli cell function in the adult male mouse^30^. Overexpression of *Sox10* results in female-to-male sex reversal in the XX mouse, and its duplication on human chromosome 22q13 causes 46, XX testicular disorders of sex development (DSD)^31^,^32^,^33^,^34^. These reports suggest that SOX8 and SOX10 might compensate for SOX9 function in male differentiation.

In this study, we investigated the mechanism of sexual differentiation in an *SRY*-absent mammal, the Amami spiny rat (*Tokudaia osimensis*). The sex chromosome constitution of this species is XO/XO, which is caused by the absence of the Y chromosome^35^,^36^,^37^,^38^. Furthermore, this species lacks the *SRY* gene^39^,^40^,^41^, suggesting that *T. osimensis* has a unique sex-determining mechanism^42^. Although SOX9 is important for the sexual differentiation of this species, the enhancer activity of *T. osimensis* TESCO is not promoted by SOX9 and SF1^43^. We report that *AMH* expression is regulated by SOX9 and SF1 in *T. osimensis*, and that ER71 regulates *SOX9* transcription, similar to that observed in the mouse. However, SOX8 and SOX10 failed to activate *T. osimensis* TESCO. Our results indicate that the mechanism of sexual differentiation following *ER71* to *AMH* expression is highly conserved in this mammal.

## Results

### Gene sequences and proximal promoter regions are conserved

The sizes of the open reading frames and the corresponding amino acids of *AMH, SOX8, SOX10*, and *ER71* of *T. osimensis* are shown in Table 1. Their nucleotide and amino acid sequences were highly similar to those of the mouse and rat (Table 1). In particular, the functional domain of each gene was highly conserved between the mouse and rat. The TGF-β domain of AMH in the mouse and rat was 98.9% and 97.0% homologous, respectively; the HMG domain of SOX8 and SOX10 was completely identical; and the ETS domain of ER71 was 98.8% and 100% homologous, respectively. The sequences of the *AMH* proximal promoter (−357/+13) and the *SOX9* promoter (−451/+13) were determined in *T. osimensis* (Fig. S1). All BSs (one SOX BS, two SF1 BSs, one GATA4 BS, and one WT1 BS) in the *AMH* proximal promoter were conserved in *T. osimensis* (Fig. S1a). Five ETS BSs (−308/−305, −292/−289, −215/−212, −170/−167, and −33/−30) were found in the *SOX9* promoter (Fig. S1b).

### FISH mapping of *AMH, SOX8,* and *SOX10*

**Figure 1:**
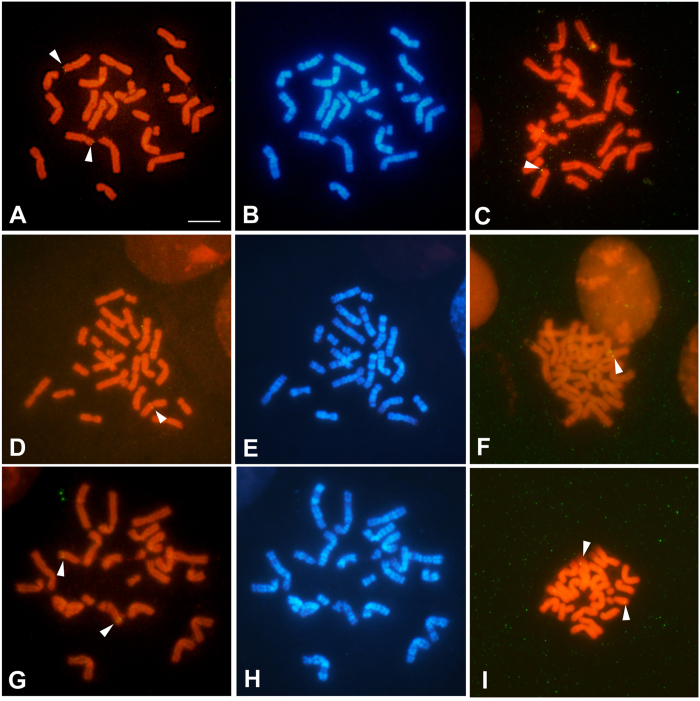
Chromosomal localization of *T. osimensis AMH, SOX8*, and *SOX10.* The *AMH* (**A**,**B**), *SOX8* (**D**,**E**), and *SOX9* (**G,I**) BAC clones, and the *Amh* (**C**), *Sox8* (**F**), and *Sox9* (**I**) cDNA clones were used as probes. Metaphase chromosomes were prepared from male *T. osimensis. AMH, SOX8*, and *SOX10* were mapped to 8p13 (**A**–**C**), 3q12 (**D**–**F**), and 10q21 (**G**–**I**), respectively. The locations of specific gene signals were identical between BAC and cDNA clones. An arrowhead marks the hybridization signal. Propidium iodide-stained R- and Hoechst G-banding patterns are shown in (**A**,**C**,**D**,**F**,**G**,**I**) and (**B**,**E**,**F**,**H**) respectively. Scale bars represent 10 μm.

To determine the chromosomal locations of *AMH, SOX8,* and *SOX10* in the *T. osimensis* genome, FISH mapping was performed. The *T. osimensis* BAC clones containing the open reading frames of *AMH, SOX8*, and *SOX10*, and the cDNA clone of each gene were used as probes. *AMH, SOX8,* and *SOX10* were mapped to 8p13 (Fig. 1A,B), 3q12 (Fig. 1D,E), and 10q21 (Fig. 1G,H), respectively, by BAC FISH. To confirm that there were no duplicated copies of these genes in other loci, we performed also FISH mapping using cDNA clone of each gene. Similarly, each cDNA clone localized to the same chromosomal location (Fig. 1C,F,I). Each probe was found at a single locus in a pair of chromosomes.

### SOX9 induces *AMH* promoter transcriptional activity

**Figure 2:**
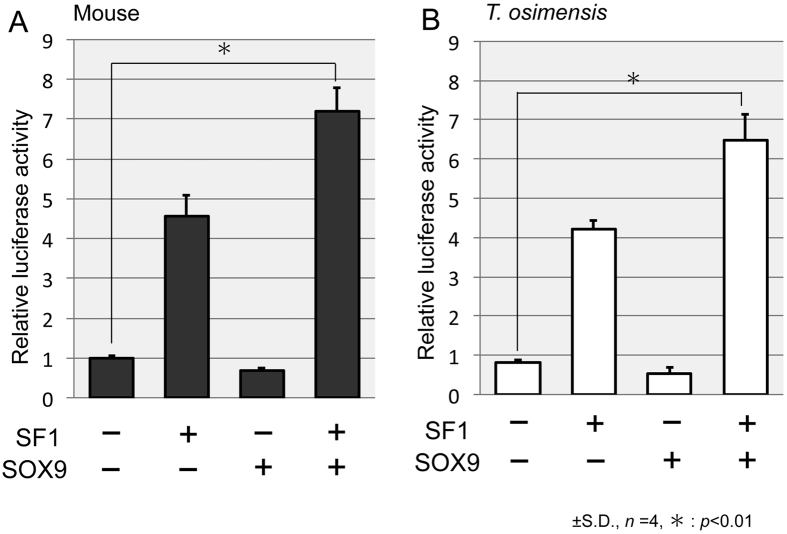
SOX9 and SF1 activate the *AMH* promoter. (**A**) Reporter gene activity of the mouse *Amh* promoter after co-transfection of SF1 and mSOX9 in Cos7 cells. A 7-fold increase in activity was observed after co-transfection with SF1 and mSOX9. (**B**) Reporter gene activity of the *T. osimensis AMH* promoter after transfection with SF1 or TOS_SOX9 in Cos7 cells. The *AMH* promoter was activated by SF1 and TOS_SOX9 in *T. osimensis*. The means ± SD from at least four independent experiments are shown.

The luciferase reporter construct (pGL3) containing the promoter region was co-transfected with the expression vector (pcDNA) into Cos7 cells. The generation of mouse *Sox9* (mSOX9), *T. osimensis* SF1 (SF1), and *T. osimensis SOX9* (TOS_SOX9) expression constructs were previously reported^43^. The luciferase vector containing the mouse *Amh* promoter (mAmh_pro) or the *T. osimensis AMH* proximal promoter (TOS_AMH_pro), and the different combinations of expression vectors were co-transfected into Cos7 cells. SF1 stimulated the activity of the positive control mAmh_pro by 4.5-fold compared to that observed for the empty vectors, whereas mSOX9 failed to up-regulate mAmh_pro activity (Fig. 2A). There was an approximately 7-fold increase in mAmh_pro activity after co-transfection with SF1 and mSOX9. These results agreed with a previous study^14^. Similarly, co-transfection with SF1 and TOS_SOX9 up-regulated TOS_AMH_pro activity (Fig. 2B).

### Mutational analysis of the *AMH* promoter in *T. osimensis*

**Figure 3:**
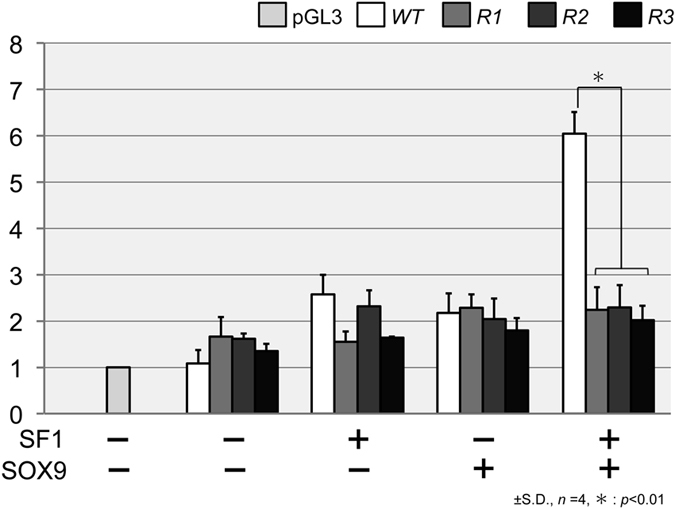
SOX9 and SF1 bind to the T. osimensis *AMH* promoter. The three *AMH* promoter mutants displayed low activity after co-transfection of different combinations of mouse and *T. osimensis* constructs. The fold-change in activity was calculated relative to the luciferase activity obtained from transfection with pGL3-empty constructs alone. *R1*, the mutation was introduced in the proximal SF1 BS within the *AMH* promoter of *T. osimensis. R2*, the mutation was introduced in the SOX BS. *R3*, the mutations were introduced in both SF1 BS and SOX BS *in cis*. The sequences of *R1, R2*, and *R3* are shown in Fig. S3. The means ± SD from at least four independent experiments are shown.

To determine whether SOX9 and SF1 bind to SOX BS and SF1 BS, respectively, to activate the *AMH* promoter, mutations were introduced in the proximal SF1 BS (*R1; Regulatory mutation-1*), the SOX BS (*R2*), and both SF1 BS and SOX BS in *cis (R3*)^22^,^27^ (Fig. S2). Mutations were introduced in TOS_AMH_pro by site-directed mutagenesis or splicing by overlap extension (SOE) PCR^44^. The mutated *AMH* promoters (*R1, R2,* and *R3*) were introduced into the pGL3 vector and co-transfected with SF1, TOS_SOX9, or both SF1 and TOS_SOX9 into Cos7 cells. The mutated TOS *AMH* promoters did not exhibit significant activity using different combinations of the expression constructs (Fig. 3).

### SOX8 and SOX10 induce the transcriptional activities of the *AMH* promoter but not TESCO in *T. osimensis*

**Figure 4:**
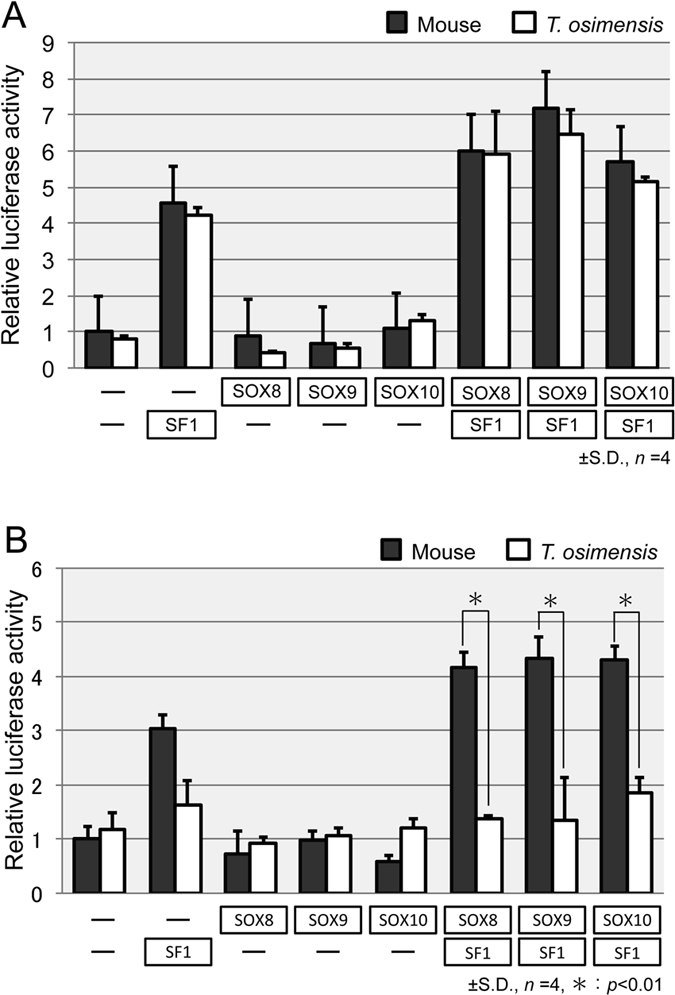
SOX8 and SOX10, but not TESCO, activate the *T. osimensis AMH* promoter. (**A**) The *T. osimensis AMH* promoter activity was enhanced by each combination of SOXE together with SF1, similar to that observed for the mouse. The fold-change in activity was calculated relative to the luciferase activity obtained from transfection with mAmh_pro alone. (**B**) *T. osimensis* TESCO displayed no enhancer activity for the different combinations of transfections. The fold-change in activity was calculated relative to the luciferase activity obtained from transfection with mTESCO alone. The means ± SD from at least four independent experiments are shown for both assays.

In the mouse, the *Amh* proximal promoter was activated by SF1 and SOX8, as well as by SF1 and SOX10 *in vitro*, similar to that observed for SF1 and SOX9^27^,^29^. We investigated whether SOX8 and SOX10 could activate the *AMH* proximal promoter with SF1 in *T. osimensis* by a reporter gene assay. The open reading frames of mouse *Sox8* and *Sox10* (mSOX8 and mSOX10), and those of *T. osimensis SOX8* and *SOX10* (TOS_SOX8 and TOS_SOX10) were cloned into the pcDNA vector. The pGL3 vector containing mAmh_pro or TOS_AMH_pro, and the different combinations of expression vectors were transiently co-transfected into Cos7 cells. The mouse *Amh* promoter showed an approximately 7-fold increase in activity when co-transfected with both SF1 and mSOX8 or SF1 and mSOX10, similar to that observed for SF1 and mSOX9 (Fig. 4A). These results agreed with a previous study^27^,^29^. Similarly, TOS_AMH_pro was significantly activated by SF1 and TOS_SOX8, SF1 and TOS_SOX10, and SF1 and TOS_SOX9 (Fig. 4A).

To determine whether SF1 and SOX8 or SF1 and SOX10 could stimulate the enhancer activity of *T. osimensis* TESCO, a reporter gene assay was performed. The pGL3 vector containing the promoter of mouse *Sox9* and mouse TESCO (mTESCO) or *T. osimensis* TESCO (TOS_TESCO), which was prepared as previously described^43^, and the different combinations of expression vectors were transiently co-transfected into Cos7 cells. SF1 alone stimulated mouse TESCO activity by 3-fold compared with that of the empty expression vector, whereas mouse TESCO was not significantly activated by mSOX8, mSOX9, or mSOX10 alone (Fig. 4B). Mouse TESCO showed a greater than 4-fold increase in activity when co-transfected with SF1 and mSOX8, SF1 and mSOX9, or SF1 and mSOX10. These results agreed with a previous study^29^. Unlike mouse TESCO, TOS_TESCO did not exhibit significant activity using all combinations of the expression constructs (Fig. 4B). The SF1-mediated activities of TESCO were limited to approximately a 2-fold increase, and SF1 and TOS_SOX8, TOS_SOX9, and TOS_SOX10 failed to activate TOS_TESCO, resulting in a 2- to 2.5-fold increase in activity as previously reported^43^.

### Expression of *SOXE* genes and *ER71* in *T. osimensis*

The expression of *Sox8*/*SOX8, Sox9*/*SOX9, Sox10*/*SOX10*, and *Er71*/*ER71* in several male and female mice and *T. osimensis* tissues was examined (Fig. S3). The expression patterns of the *SOXE* genes were mostly consistent with that of the mouse (Fig. S3A). However, testis-specific *Er71*/*ER71* expression was observed in both the mouse and *T. osimensis* (Fig. S3B).

### ER71 induces the transcriptional activity of the *SOX9* promoter

**Figure 5:**
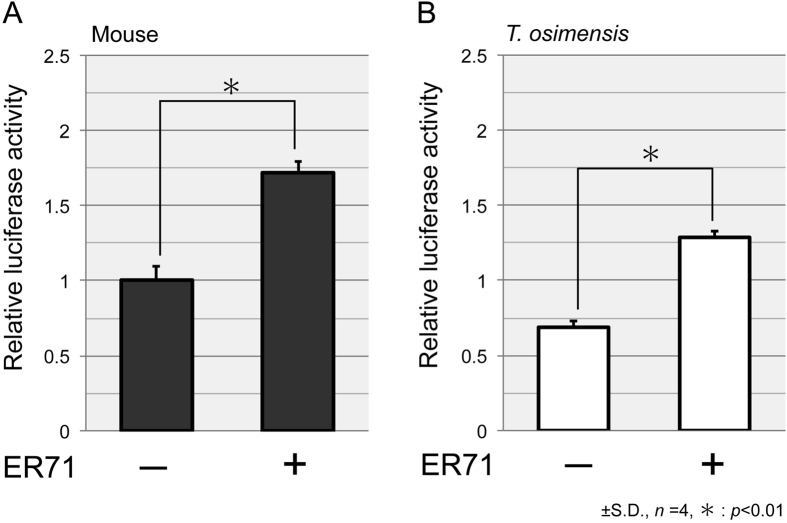
ER71 activates the *SOX9* promoter. Mouse (**A**) and *T. osimensis* (**B**) *ER71* activates the *Sox9*/*SOX9* promoter. The fold-change in activity was calculated relative to the luciferase activity obtained from transfection with mSox9*_*pro alone. The means ± SD from at least four independent experiments are shown.

A reporter gene assay was performed to determine whether ER71 can enhance the activity of the *SOX9* proximal promoter in *T. osimensis*. The luciferase vectors containing the −453/+13 *SOX9* proximal promoter of *T. osimensis* (TOS_SOX9_pro) or that of the mouse (mSOX9_pro), and the pcDNA containing the *Er71* open reading frame of the mouse (mER71) or that of *T. osimensis* (TOS_ER71) were transiently co-transfected into Cos7 cells. For the positive control, the *Sox9* promoter showed an approximately 2-fold increase in activity when co-transfected with ER71 expression constructs (Fig. 5A). TOS_*SOX9*_pro was also activated by ER71 in *T. osimensis* (Fig. 5B).

## Discussion

The nucleotide and amino acid sequences of *T. osimensis AMH, SOX8, SOX10*, and *ER71* were highly similar with those of the mouse and rat (Table 1). In addition, the functional domain of each gene in *T. osimensis* was highly homologous with that of rodent genes. Results from FISH mapping revealed that each gene existed as a single copy within the genome (Fig. S2), indicating evolutionary conservation in this species.

The *AMH* proximal promoter sequence was highly conserved in *T. osimensis* (Fig. S1A). The reporter gene assays showed each SOXE protein stimulated the activity of *AMH* promoter together with SF1 like mouse (Fig. 4A), indicating that *SOXE* genes might function in sexual differentiation in male spiny rats. To determine whether SOX9 and SF1 bind to SOX BS and SF1 BS, respectively, and activate the *AMH* promoter, we performed reporter gene assays using three *AMH* promoter mutants of SOX BS and proximal SF1 BS (*R1, R2*, and *R3*, Fig. S3). Promoter mutants significantly reduced the luciferase activity (Fig. 3), revealing that binding of SOX9 and SF1 to BS is essential for the regulation of *AMH* expression. These results confirmed that the regulation of *AMH* by *SOXE* genes such as SOX9, which is especially important, was conserved in *T. osimensis*.

By contrast, TESCO enhancer activity was not stimulated by the *SOXE* genes and SF1 (Fig. 4B). This result was consistent with a previous study that demonstrated loss of TESCO enhancer activity in *T. osimensis, T. tokunoshimensis*, and *T. muenninki*^43^. The loss of enhancer activity was caused by nucleotide substitutions of *SRY* BS and SF1 BS within TESCO, leading us to conclude that SOX8 and SOX10 failed to activate TESCO due to substitutions. Indeed, *SRY* was lost in *T. osimensis* and TESCO displayed no enhancer activity, whereas *SOX9* was expressed in the testes (Fig. S3). Our results support an idea that *SOX9* expression in the testes must be regulated via another enhancer in *T. osimensi*^43^. In human, 516–584 kb upstream duplication and 607.1–639.6 kb upstream deletion of *SOX9* cause XX DSD in the absence of *SRY* and XY DSD, respectively^45^. These discoveries implying the existence of other enhancers that work in concordance with a testis-specific enhancer such as TESCO and/or other regulatory elements for the gonad-specific expression pattern of *SOX9*.

Five ETS BSs were identified in the *SOX9* proximal promoter of *T. osimensis*, and three out of five were species-specific (−215/−212, −170/−167, and −33/−30; Fig. S1B). Results from the reporter gene assay showed ER71 to enhance *SOX9* promoter activity, illustrating the function of the *SOX9* promoter was conserved in this species (Fig. 5B). In addition, *ER71* was expressed in *T. osimensis* testes (Fig. S3). These results indicated that *ER71* expression is regulated by SOX9, and that the downstream molecular pathway of *ER71* is highly conserved in *T. osimensis* in the absence of *SRY* expression.

In the mouse, SP1 binds to the *Sry* promoter to activate *Sry* transcription^46^,^47^. SP1 is a zinc finger transcriptional factor ubiquitously expressed^48^,^49^. *SRY* enhances the *Er71* expression by binding with SP1 to its promoter region^10^. In this study, *ER71* expression was detected in the *T. osimensis* testis, suggesting that another *ER71*-regulated gene has superseded the function of *SRY*. There is a possibility that SP1 and other SOX genes such as SOX3, SOX8, and SOX10 may trigger *ER71* expression in fetal gonads. However, additional studies are needed to clarify the regulation of ER71 expression and to identify the new sex-determining gene in *T. osimensis*.

**Figure 6:**
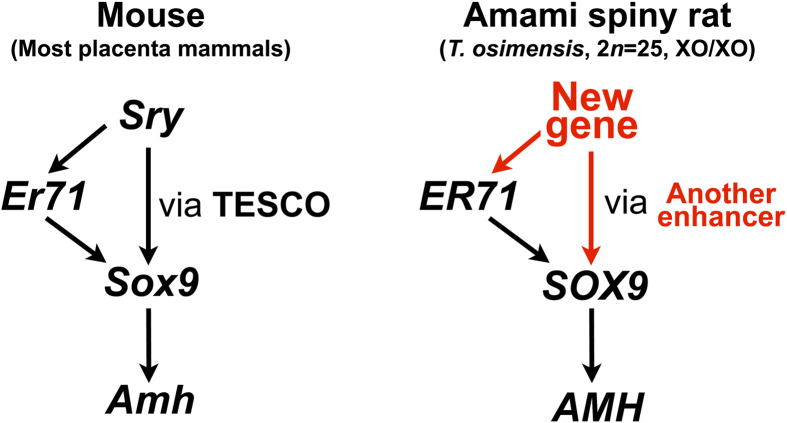
Schematic model for sexual differentiation in the *SRY*-absent mammal, *T. osimensis*. In the male Amami spiny rat (*T. osimensis*), a new sex-determining gene superseded *SRY*. This gene might activate *SOX9* via another enhancer (not TESCO) and *ER71* during sexual differentiation. This study showed that the downstream cascade of *SOX9* was conserved in this species.

On the basis of several reports and theoretical considerations, the evolution of sex-determining genes is believed to proceed from less to more complex^50^,^51^, suggesting that molecular regulation of downstream genes are more highly conserved between taxonomic groups. Our results, which showed that the regulations of *SOX9* by *ER71* and *AMH* by *SOX9* were highly conserved in the *SRY*-absent species (Fig. 6), support this contention. In conclusion, we showed that the molecular cascades involved in male sexual differentiation are highly conserved in the *SRY*-absent species. These findings contribute to evolutionary studies of sex-determining and sex-differentiating genes in eutherian mammals.

## Materials and Methods

### Animals

*T. osimensis*, an endangered species (The IUCN Red List of Threatened Species; http://www.iucnredlist.org/1/1/2016), has been protected by the Japanese government as such since 1972. With permission from the Agency for Cultural Affairs, the Ministry of the Environment in Japan, *T. osimensis* were captured in cage traps on Amami-Oshima Island. To obtain fibroblasts for cell culture experiments, the tips of their tails were cut with surgical scissors. Tissues were harvested from animals that died naturally or accidentally. Total RNA was obtained from two females and two males. All the animal experiments in this study were approved by the Institutional Animal Care and Use Committee of the National University Corporation Hokkaido University and performed in accordance with the Guidelines for the Care and Use of Laboratory Animals, Hokkaido University.

### Isolation of BAC clones containing *AMH, SOX8*, and *SOX10*

A *T. osimensis* BAC library was previously constructed^43^. PCR primer pairs were designed (Table S1) and used to screen the BAC library using a two-step 3D PCR screening system as previously described^43^. The isolated BAC clones of *AMH, SOX8*, and *SOX10* were defined as TOB1-73N22, TOB1-283L22, and TOB1-65I6.

### Cloning and sequencing of each gene and promoter

Total RNA was extracted from mouse and *T. osimensis* tissues using the RNeasy Mini Kit (QIAGEN) according to the manufacturer’s instructions. The total RNA was reverse transcribed using SuperScript III (Invitorgen) and oligo(dT) primers. The synthesized cDNA and BAC clones were used as templates for coding sequence (CDS) amplification and promoter sequence amplification, respectively. We designed primer pairs to amplify the coding regions of *AMH, SOX8, SOX10* and *ER71* and the promoter regions of *AMH* and *SOX9* by comparing mouse and rat DNA sequences. The primer sequences are shown in Table S1. The GenBank accession number of each gene is as follows: LC149849 for *AMH* CDS, LC149850 for *SOX8* CDS, LC149851 for *SOX10* CDS, LC149852 for *ER71* CDS, LC149853 for the AMH promoter, and LC149854 for the *SOX9* promoter.

### Preparation of chromosomes for FISH mapping

The R-banded chromosomes and BAC FISH were prepared as previously described^43^. FISH using cDNA probes was performed as earlier described^38^.

### Construction of plasmids for promoter analysis

The pcDNA3.1 (+) (Invitrogen) expression vector was used to prepare the plasmids. The entire open reading frame of each gene was cloned into the *Hind* III/*BamH* I restriction sites of the expression vector. The expression vectors inserted *Sox9*/*SOX9* (mSOX9 and TOS_SOX9) and *SF1* were previously constructed^43^. The amino acid sequence of mouse and *T. osimensis* SF1 was identical; therefore, we used *T. osimensis SF1* expression vectors in all experiments. The *AMH* promoter (−357 to +13) and the *SOX9* promoter (−451 to +13) were ligated into the *Xho* I/*BamH* I restriction sites of the pGL3-basic vector (Promega). To generate a mutant *AMH* promoter reporter construct, which would have mutations in the SOX or SF1 BS as previously described^22^, site-directed mutagenesis and SOE PCR were performed^44^. The sequence of each primer is shown in Table S1.

### Reporter gene assays

COS7 cells were cultured in DMEM supplemented with 10% fetal bovine serum at 37 °C in an atmosphere of 5% CO_2_. COS7 cells were seeded at a density of 0.5 × 10^5^ per well in a 24-well plate 24 h prior to transfection. Transfection was performed using 1.5 μl of Lipofectamine 3000 (Invitorogen). To measure the activity of TESCO, the reporter construct (550 ng of pGL3_mTESCO_SOX9pro, mTESCO, pGL3_TOSTESCO_SOX9pro, or TOSTESCO), different combinations of the expression vector (110 ng) or the pRL *Renilla* luciferase control reporter vector (30 μg) (Promega) were transfected according to the manufacturer’s instructions. The quantity of the expression vector was increased to 220 ng with the empty pcDNA3.1 vector. The activity of the *AMH* promoter was measured by using the reporter construct (400 ng of pGL3_mAMHpro, mMMHpro, pGL3_TOSAMHpro, TOSAMHpro, pGL3_TOSAMHpro_ SF1BSmutated [*R1*], pGL3_TOSAMHpro_SOXBSmutated [*R2*], or pGL3_TOSAMHpro_ SF1BS/SOXBSmutated [*R3*]), different combinations of the expression vector (20 or 40 ng), and pRL (20 ng). The quantity of the expression vector was increased to 60 ng with the empty pcDNA3.1 vector. To measure activity of *SOX9* promoter, either 430 ng of reporter construct (pGL3_MSOX9pro or mSOX9pro, pGL3_TOSSOX9pro or TOSSOX9pro), several combinations of 43 ng of each expression vectors, and 20 ng of pRL. The total amount of expression vector was adjusted to 43 ng by empty pcDNA3.1. Forty-eight hours after transfection, the reporter activities were measured by Dual-Luciferase Reporter Assay System (Promega) according to the manufacturer’s instructions. The reporter activity was normalized to Renilla luciferase activity as an internal control. Each experiment was carried out four independent times.

## Additional Information

**How to cite this article**: Otake, T. and Kuroiwa, A. Molecular mechanism of male differentiation is conserved in the *SRY*-absent mammal, *Tokudaia osimensis. Sci. Rep.* **6**, 32874; doi: 10.1038/srep32874 (2016).

## Supplementary Material

Supplementary Information

## Figures and Tables

**Table 1:** Sequence identity among mouse, rat and *T. osimensis.*

Gene (size)	Mouse (%)	Rat (%)
*AMH* (1,665 bp)	93.3	91.7
AMH (554 aa)	92.2	91.5
*SOX8* (1,395 bp)	96.2	95.6
SOX8 (464 aa)	98.7	97.8
*SOX10* (1,401 bp)	96.8	96.2
SOX10 (466 aa)	99.8	98.7
*ER71* (1,008 bp)	95.1	93.5
ER71 (335 aa)	94.3	91.7
