# Supplementary material for: Molecular mechanism of male differentiation is conserved in the SRY-absent mammal, Tokudaia osimensis
Source: Sci Rep. 2016 Sep 9;6:32874. doi: 10.1038/srep32874 (PMC5017195; doi:10.1038/srep32874)
Supplement: Supplementary Information [file srep32874-s1.pdf]

Supplementary Information

Molecular mechanism of male differentiation is conserved in the *SRY*-absent mammal, *Tokudaia osimensis*

Tomofumi Otake[1] & Asato Kuroiwa[1, 2]

[1] Functional Genome Science Biosystems Science Course, Graduate School of Life Science, Hokkaido University, Kita 10 Nishi 8, Kita-ku, Sapporo, Hokkaido 060-0810, Japan

[2] Division of Reproductive and Developmental Biology, Department of Biological Sciences, Faculty of Science, Hokkaido University, Kita 10 Nishi 8, Kita-ku, Sapporo, Hokkaido 060-0810, Japan

Tel/Fax: +81-11-706-2752; E-mail: asatok@sci.hokudai.ac.jp

Correspondence: A. Kuroiwa

Supplementary Figure Legends

Figure S1. Sequence alignment of the mouse, rat, and *T. osimensis AMH* proximal promoter

(A) Sequence alignment of the *AMH* proximal promoter. The SF1 BS, SOX BS, GATA4 BS, and WT1 BS are boxed with black, red, blue, and gray squares, respectively. +1 marks the transcriptional start site in the mouse. (B) Sequence alignment in the *SOX9* proximal promoter. The ETS BS sequences (5'-GGAA/T-3', 5'-T/ATCC-3' in reverse) are denoted in gray. The number of the nucleotide corresponds to the *T. osimensis* sequence.

Figure S2. Mutagenesis of the *T. osimensis AMH* promoter

Nucleotide substitutions were introduced in the SF1 BS (*R1*, *regulatory mutation-1*), SOX BS (*R2*, *regulatory mutation-2*), and both SF1 BS and SOX BS *in cis* (*R3*, *regulatory mutation-3*). These mutations were reported in previous reports for mouse[22, 27].

Figure S3. Expression of *Sox9/SOX9*, *Sox8/SOX8*, *Sox10/SOX10* and *Er71/ER71* in mice and *T. osimensis*

The expression patterns of *Sox9/SOX9, Sox8/SOX8, Sox10/SOX10* (A) and *Er71/ER71* (B) in male and female mice and *T. osimensis* by RT-PCR. H, heart; Li, liver; K, kidney; S, spleen; B, brain; Lu, lung; T, testis; O, ovary. *Actb/ACTB* was used as the internal control. For the negative control, distilled sterile water (dH_2O) was used as the template.

A

```
Mouse         -357   TTTCT ATTT- GTCCT GCCTT GAGCC TATTA AACAG CCTCC CCCGT GTCTC CCTGT CTGC-   -298
Rat                  TTTCT ATTTT GTCCT GTCCT AAGTC AATTA AACAG CCTCC CCCAT GTCTC CCTGT CTGCA
T. osimensis         TTTCT ATTTT GTCCT GTTCT TTGCC TATTA AACAG CCTCC CCCAT GTCTC CCTGT CTGCA

Mouse         -297   -TGTG TGTTT --GGT AGTGG GGA-G GGTGG ATACT GCTTG TCTCT GTTGA AGCTG TGGTG   -240
Rat                  GTGTG TGTTT TTGGT AGTGG GGTTG GGTGG GTACT GCTTG TCTCT ATGGA AGGCG TGGTG
T. osimensis         GTGTG TTTTT --GGT AGTGG GGATG GGTGG GTACT GCTTG TCTCT GTTGA AGGCG TGGTG
                                                                          Distal SF1 BS
Mouse         -239   ACCTG GGGCG TCCTC CAGGT GGGCT CCCCA GGGAG ATGGG AGCTA CTCAA GGACA GCTCA   -183
Rat                  ACCTG GGGGA TCGTC CAGGT GGGCT CCCCA GGCAG ATGGG AACGA CCCAA GGACA CCTC-
T. osimensis         ACCTG GGG-- TCTTC CAGGT GGGCT CCCCA GGG-G ATGGG AGCTA CTCAA GGACA GCTCT
                                                                  SOX BS
Mouse         -182   GGCCT CTGCA GTTAT GGGCC CAGCT CTGAG GACAG AAAGC CCTTT GAGAC AGTCG CCTCC   -123
Rat                  GGCCT CTGCA GTTG- GGGGC TGGCT CTGAG GACAG AAAGC CTTTT GAGAC AGCCG CCTCC
T. osimensis         GGCCT CTGCA GTTGT GGGCC CAGCC CTGAG GACAG AAAGC CCTTT GAGAC AGCCG CCTCC
                                                    Proximal SF1 BS                  GATA4 BS
Mouse         -122   CACCT GCTGG GCATG AAAAG TGC-- -CAGG CACTG TCCCC CAAGG TCACC TTTGG TGTTG    -66
Rat                  CACCT GATGG GCATG CAAAG CGCAG CCAGG CACTG TCC-C CAAGG TCACC TCAGG GGTTG
T. osimensis         CACCT GCTGG GCATG CAGAG CGC-- -CAGG CACTG TCCCC CAAGG TCACC TCTGG AGTTG
                                                                                WT1 BS
Mouse          -65   ATAGG GGCGT CCCTC CCAAG CAAGC AATCT GGCTC AGCCA TACAT ATAAG CAGGG CCACC    -10
Rat                  ATAGA GGTGT CCCTC CCAAG CAC-- --TCT GGCTC GA--A TGCAT ATAAG CAGGA CCACC
T. osimensis         ATAGG GGTGT CCCTC CCAAG CAC-- --TCT GGCTC AGCCA TACAT ATAAG CAGGG CCACC
                               +1
Mouse           -9   CGGAC CTTGC TGTAC CACCA TG    +13
Rat                  CAGAC CCTGC CATGC CACCA TG
T. osimensis         CAGAC CTTGT TGTAC CACCA TG
```

B

```
Mouse         -451   CATCG AAAAG TGGGG GTGGG GGGTT ---GT GGAGG GTCCT AGTCT AGACA CGCTC GCGTG   -392
Rat                  TAGTG GACTG TGGGT GGGGG GGGGC ---GT GGAGG ATAGT GGG-- AGGGG GGTCC TAGTC
T. osimensis         CATCG AAAAG TAGGG GGGGG GGGAG TGGGA GGGGG ACCCT AGTCT AGACA CGCGC CCGTG

Mouse         -391   CACGC GCACA CACAC ACACA CACAC ACACA TACAC ACACA CACAC ACACA CACAC ACACA   -331
Rat                  TAGAC ATGCG CGCGT ACACA CACAC ACACA CACAC ACACA CATCA GCACA CACAC -TACA
T. osimensis         CACGC ACGCG CGCGC GCGCA CACAC ACACA CACAC AGACA CACAC ACACA CACAC ACACA
                                                    -308 -305              -292 -289
Mouse         -330   CACAC ACATC GGTTC ACACG GAGAC C-GTT CCAAA ACTGT GACAT TCCGA GAGTA GAGAG   -277
Rat                  CAC-- ----- ---AC ACAAG GAGAC --GTT CCAAA ACTGT GACAT TCGGA GAGTA GAGAG
T. osimensis         CAC-- ----C GATTC ACACG GAGAC CCGTT CCAAA ACTGT GACAT TCCGA GAGTA GAGAG

Mouse         -276   CAAAC TTACA CACTC GGACG TCCCG GGTCC CCCGC TTGCC CCGCG CCCCC CTC-- --CAA   -217
Rat                  CAAAC TTACA CACTC GGACG TCCCG GGTCC CCCGC CTGCC CCGCG CCCCC CCCCC --CAA
T. osimensis         CAAAC TTACA CACTC GGACG TCCCG GGTCC CCCGC CTGCC CCGCG CCCCC CTCCC TCCAA
                   -215 -212                                                     -170 -167
Mouse         -216   GTCCC CTCAC CCCAC CATCC ----- -ACCC TCTGG CTGAG CTCCC CTCCC ---TT CTCCT   -163
Rat                  GTCCC CTCAC CCCAC CATCC CCATC CACCC TCCGG CTGAG CTTCC CTCCC ---CT CTCCT
T. osimensis         GTTCC CTCAC CCCAC CATCT ----- -ACCC TCCGG CCGAG CTCCC CCCCC CCTTC CTTCT
                                                           -131 -128
Mouse         -162   CCCCT CCTGT CTC-- --GTC ACCCA ACCCG GAGCC ACAAT CCTCC CCAGC CCCCC TTCCA   -110
Rat                  CCCCT CGAGT CTC-- --GTC ACCCA ACCCG GAGCC ACAAT CCTCC CC--C CACCC TTTCA
T. osimensis         CCTCC CTTGT GTCTC GTGTC GCCCA ACCCG GAGCC ACAAT TCTCG CC--- ----C CTTCA

Mouse         -109   AAATC CGGTC CAATC AGCGA CTTGC CAACA CTGAT GACTC AAGAG CTAGC CGTGA TTGGC    -53
Rat                  AAATC CGGTC CAATC AGCGA CTTGC CAACC CTGAT GACTG CCGA- ---GC CGTGA TTGGT
T. osimensis         AACTT GAGTC CAATC AGCGA CTTGC CAAGC CCCTT GACTG ACCGA TTGGC CGAGG T---C
                                             -33  -30                              +1
Mouse          -52   CCGAG GTATC TAACG TGAAG GAGGA GTATT TATTA --GAG ACCCT GAGCT GGAAG TCGGA     +2
Rat                  CCGAG GTCTC TAAGG TGAAG GGGGA GTATT TATTA AAGAG ACTCT GGGCT GGGAG CCGGA
T. osimensis         TCGGC GTG-- -AAGG AGGAG GAGGA TT-TA TAATA --GAG ACCCT GGGCT GGGAG TCGGA

Mouse           +3   GAGCC GAGAG C   +13
Rat                  GAGCT GAGAG C
T. osimensis         GAGCC GAGAG C
```

Fig S1

	SOX BS	proximal SF1 BS
**WT**	5′-CCCTTTGAGA-3′	5′-CCAAGGTCA-3′
R1	5′-CCCTTTGAGA-3′	5′-CCAATTTCA-3′
R2	5′-ACCACACGTC-3′	5′-CCAAGGTCA-3′
R3	5′-ACCACACGTC-3′	5′-CCAATTTCA-3′

Fig S2

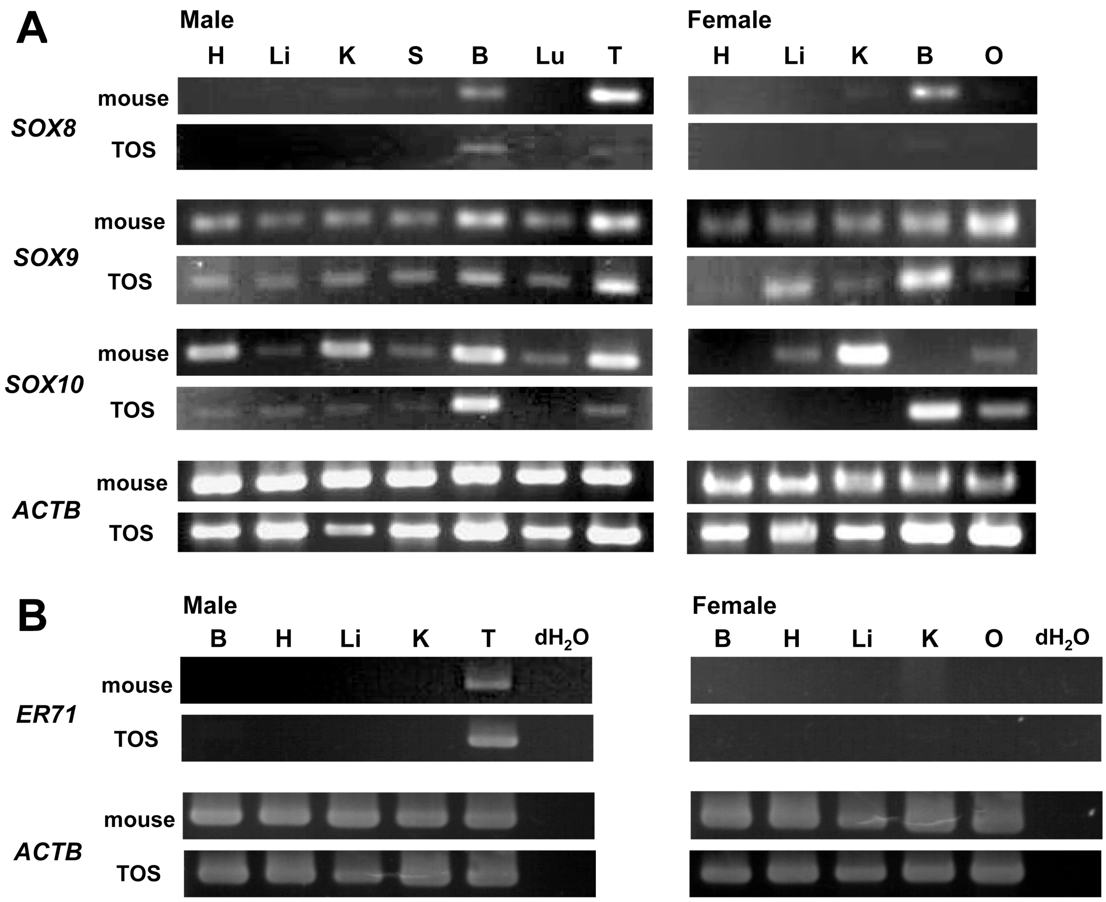
A
Male
H Li K S B Lu T
Female
H Li K B O
SOX8
mouse
TOS
SOX9
mouse
TOS
SOX10
mouse
TOS
ACTB
mouse
TOS
B
Male
B H Li K T dH2O
Female
B H Li K O dH2O
ER71
mouse
TOS
ACTB
mouse
TOS

Fig S3
